# Pathogen detection in the CRISPR–Cas era

**DOI:** 10.1186/s13073-018-0543-4

**Published:** 2018-04-24

**Authors:** Dipali G. Sashital

**Affiliations:** Roy J. Carver Department of Biochemistry, Biophysics & Molecular Biology, Iowa State University, Ames, IA 50011 USA

**Keywords:** CRISPR–Cas, Pathogen detection, Diagnostics, Genotyping, Biotechnology, Virus, Bacteria

## Abstract

CRISPR–Cas systems have provided revolutionary tools for genome editing. The discovery of Cas proteins with alternative activities has now enabled sensitive and robust tools for detecting nucleic acids. Recent reports harnessing these new CRISPR–Cas technologies display their potential for providing low-cost and practical diagnostic tools for pathogen and disease detection.

## Collateral cleavage is an alternative activity of several Cas proteins

Prokaryotic CRISPR–Cas (clustered regularly interspaced short palindromic repeats–CRISPR-associated) immune systems have offered a treasure trove of biotechnological tools over the past 5 years [1]. Most of these technologies have relied on the RNA-guided activity of Cas proteins to target and cleave specific nucleic acid sequences. However, the recent discovery of non-canonical activities in some Cas proteins has opened the door to new tools that offer cost-effective, portable diagnostics through nucleic acid sensing [2–4].

**Fig. 1:**
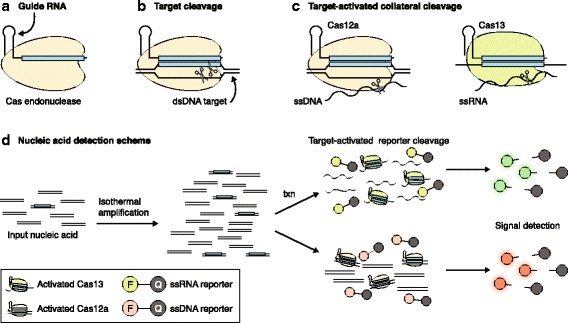
Overview of Cas endonuclease activity and nucleic acid detection systems. **a** Cas endonucleases are single protein effectors for guide RNAs. **b** Cas endonucleases bind targets complementary to target DNA (Cas9 or Cas12) or RNA (Cas13) and cleave the target (Cas9 or Cas12). **c** For Cas12a and Cas13, target binding also activates collateral cleavage of single-stranded DNA (*ssDNA*; Cas12a) or single-stranded RNA (*ssRNA*; Cas13) supplied in *trans*. **d** This activity can be exploited for nucleic acid detection. For attomolar sensitivity, isothermal amplification of input nucleic acid is required. The resulting DNA can be transcribed (*txn*) for Cas13-based detection, or detected directly by Cas12a. Reporter ssRNA or ssDNA are cleaved by Cas13 or Cas12a, respectively, producing a fluorescent signal. *dsDNA* double-stranded DNA

The origins of CRISPR–Cas systems as bacterial and archaeal defense mechanisms have led to an enormous diversity of Cas proteins owing to co-evolution between host cells and their invaders [5]. The majority of CRISPR–Cas systems found in bacteria and archaea are characterized by large, multi-subunit complexes that carry out host defense (class 1), but a small subset of bacteria contains systems that require only a single protein for RNA-guided cleavage of foreign nucleic acids (class 2) (Fig. 1a). Although rarer, class 2 Cas endonucleases are readily adapted for biotechnology because of their simplicity. Cas9, the mostly commonly used Cas endonuclease, utilizes a guide RNA to bind to a complementary DNA sequence, which is subsequently cleaved through Cas9 endonucleolytic activity (Fig. 1b) [6]. Cas9 and its guide can be deployed as a two-component system for a variety of biotechnological purposes, which has exponentially expanded the accessibility of genome editing in recent years.

The discovery of two additional class 2 Cas endonucleases, Cas12a (formerly Cpf1) and Cas13a (formerly C2c2), has provided orthogonal tools for programmable double-stranded DNA and RNA targeting, respectively [1]. Surprisingly, along with their divergent sequences, Cas12a and Cas13a also deviate in their catalytic activities in comparison to Cas9 (Fig. 1c). Upon binding to a target RNA, Cas13a transforms into a nonspecific endoribonuclease that can degrade single-stranded RNA sequences supplied either in *cis* with the target or in *trans* [7]*.* This so-called collateral cleavage activity diverged from the known activity of the other class 2 Cas endonucleases, which were thought to cleave only at specific sites within the target. However, a new mechanistic study shows that Cas12a-target binding also triggers nonspecific collateral cleavage, this time against single-stranded DNA supplied in *trans* (Fig. 1c) [3]. These unexpected activities of both Cas12a and Cas13a highlight the diversity of CRISPR–Cas systems, and offer new opportunities for Cas endonuclease-based tool development.

## Exploiting target-activated collateral cleavage for nucleic acid detection

Target-activated nonspecific nucleases provide a robust mechanism for host defense, because Cas endonucleases can first sense invasive nucleic acid through specific target recognition and then amplify the signal through promiscuous collateral cleavage activity [7]. Similarly, the promiscuous activity can also be exploited to amplify target detection through the collateral cleavage of a reporter nucleic acid (Fig. 1d). Owing to the long-term and multiple turnover nature of collateral cleavage [8], the signal would be amplified over time to ensure its detection even in the presence of a small amount of the target sequence. This theory underlies the use of Cas endonucleases exhibiting collateral cleavage for nucleic acid detection.

In early work aimed at developing a Cas13a-based RNA detection tool, East-Seletsky et al. [8] demonstrated that Cas13a-target recognition could be read out by using a reporter RNA labeled with both a fluorophore and quencher. Upon Cas13a-target binding, collateral cleavage of the reporter RNA results in release of the fluorophore from the quencher and an increase in fluorescent signal (Fig. 1d). In this early incarnation, Cas13a-based detection could be used to distinguish target sequences of concentrations in the picomolar range within complex mixtures of RNA, including sensing endogenous transcripts within total cellular RNA [8].

Cas13a-based RNA detection opens the possibility of diagnostic applications through the sensing of nucleic acids associated with pathogens or diseases. However, useful diagnostic tools must be sensitive enough to detect very small amounts of nucleic acids, down to the attomolar range. To overcome the limited sensitivity of initial RNA detection tools, Gootenberg et al. [2] developed SHERLOCK (Specific High-sensitivity Enzymatic Reporter UnLOCKing), which combines isothermal amplification of nucleic acid sequences with a Cas13a-based detection platform (Fig. 1d). Isothermal amplification techniques work at a constant and low temperature, avoiding the need for the expensive equipment required for standard polymerase chain reaction methods. This allows for portable diagnostic tools that can easily be deployed in field conditions where laboratory equipment is not readily available.

By coupling isothermal amplification to Cas13a target-activated reporter cleavage, SHERLOCK enabled detection of viral and bacterial nucleic acids with attomolar sensitivity [2]. Importantly, with careful design of the Cas13a guide RNA, the authors demonstrated that SHERLOCK could distinguish between very closely related sequences with as little as one nucleotide difference. Thus, SHERLOCK can be used to discriminate between different strains of the same virus, including the African and American strains of Zika, or to genotype single-nucleotide polymorphisms in the human genome, including cancer-causing mutations in *EGFR* and *BRAF* genes.

## Second-generation CRISPR–Cas diagnostics

The successful development of CRISPR–Cas tools has relied on an understanding of the mechanisms underlying the function of Cas endonucleases. Continued investigations into Cas endonuclease mechanisms have led to substantial expansions and improvements of nucleic acid detection platforms. In a recent report, Chen et al. [3] discovered that Cas12a displays collateral cleavage against single-stranded DNA, and they capitalized on this discovery by developing DETECTR (DNA Endonuclease Targeted CRISPR *Trans* Reporter), which couples isothermal amplification with Cas12a-based DNA reporter cleavage (Fig. 1d). Using this system, the authors were able to detect two distinct genotypes of human papillomavirus in patient samples with high accuracy.

Further investigation of Cas endonuclease activity has also facilitated substantial improvements to SHERLOCK. In a follow-up to their initial study, Gootenberg et al. [4] performed extensive enzymatic analysis of several Cas13a (and the related Cas13b) orthologs, determining their sequence preferences for collateral cleavage. This characterization, along with the discovery of Cas12a collateral cleavage [3], enabled four-channel sample multiplexing, one of the key features of the updated SHERLOCKv2 platform. Using four separate reporter sequences, each exploiting the orthogonal sequence or nucleic acid specificities of four Cas13 and Cas12a variants, the authors demonstrated that SHERLOCKv2 could faithfully detect multiple RNA and DNA sequences within a single sample [4].

Potential tools for CRISPR–Cas-based diagnostics are not limited to the class 2 systems. The recent discovery that the nonspecific RNase Csm6 is activated by linear or cyclic poly-adenylate (polyA) molecules that are produced upon target-recognition by the class 1 Csm complex draws clear parallels to target-activated collateral cleavage by Cas12a and Cas13 [9, 10]. Gootenberg et al. [4] took advantage of poly(A)-activation by Csm6 to boost the signal output for SHERLOCKv2. Using a dual-reporter system, in which Cas13a cleaves a poly(A) reporter, the product of which activates Csm6 to cleave the second reporter, the authors were able to increase the signal of SHERLOCKv2 by up to 3.5-fold. Combining this improved sensitivity with a lateral-flow readout, the authors developed a paper-based test for the presence of viral RNA. In this format, a paper strip is dipped into a liquid sample, and a band appears at different locations on the strip in the absence or presence of the RNA. These results demonstrate the potential of SHERLOCK as a cost-effective alternative for rapid diagnostics in clinical settings [4].

## Conclusions and future directions

The early successes of CRISPR–Cas diagnostic tools present an exciting outlook for the future of this technology. The simplicity with which these tools can be reprogrammed makes them readily configurable for a huge variety of applications. Successful deployment of SHERLOCK in a paper-based lateral-flow format also demonstrates its capacity for easy application without the need for technical expertise or expensive equipment, similar to home pregnancy tests. However, it remains to be seen how effective these tools will be in clinical situations, especially in field conditions that could vary wildly from the laboratory settings in which the tools were developed. The use of RNA reporters for Cas13-based detection could present a potential limitation, because RNA is relatively unstable and susceptible to cleavage by cellular RNases. This could result in false positive results when using samples prepared in non-laboratory settings. It is possible that Cas12a-based diagnostics such as DETECTR [3] may be less susceptible to such potential issues, owing to the relative stability of the DNA reporter. In either case, rigorous clinical testing, including benchmarking against existing diagnostic tools, will be required to ensure the quality of results obtained through these tests.

The discovery of new CRISPR–Cas systems has provided exciting opportunities for innovation over the past few years. Continued investigation of newly discovered systems will undoubtedly uncover more useful enzymes that may be implemented for improved sensitivity or stability of the current diagnostic tools. Nevertheless, the tools may already be mature enough for implementation and clinical testing. The potential to use these types of tests for rapid diagnostics could have an enormous impact in point-of-care settings, including early detection of viral outbreaks to ensure timely public health response. As with genome editing, these new CRISPR–Cas diagnostic tools are poised to revolutionize the accessibility of rapid, sensitive and accurate diagnosis of infectious and genetic diseases for people around the world.

## Abbreviations

Cas: CRISPR associated
CRISPR: Clustered regularly interspaced short palindromic repeats
DETECTR: DNA Endonuclease Targeted CRISPR *Trans* Reporter
Poly(A): Poly-adenylate
SHERLOCK: Specific High-sensitivity Enzymatic Reporter UnLOCKing
